# Professionals’ digital competences and user profiles in social agencies and their impact on professional practice, family autonomy and wellbeing

**DOI:** 10.3389/fpsyg.2024.1363444

**Published:** 2024-03-20

**Authors:** Sonia Byrne, Guacimara Rodríguez, Míriam Álvarez, Nauzet Gutiérrez-Rodríguez, María José Rodrigo, Sonia Padilla, Juan Carlos Martín

**Affiliations:** ^1^Department of Developmental and Educational Psychology, University of La Laguna, Tenerife, Spain; ^2^Department of Social Work and Social Services, University of La Laguna, Tenerife, Spain; ^3^HESTIA Association for Family, Psychoeducational and Social Research and Intervention, Canary Islands, Spain; ^4^Department of Education, University of Las Palmas de Gran Canaria, Las Palmas, Spain

**Keywords:** digital competences, professional practice, positive parenting, family wellbeing, social agencies

## Abstract

**Introduction:**

The health crisis of the last 3 years has revealed the weaknesses of the child and family support system based on the professional use of digital resources in social agencies. This study addresses three aims: to examine the level of professional digital competence; to analyze the user profiles in a variety of digital resources; and to test the impact of level of competences and user profiles on four aspects: professional practice, family satisfaction with the services, child and family wellbeing, and family autonomy in the exercise of the parenting role.

**Methods:**

Participants were 148 practitioners from social agencies who voluntarily responded to an online survey with 47 questions.

**Results and discussion:**

Results showed that professionals perceive themselves as more competent in areas of information / data management and communication / collaboration than in the creation of digital content, security measures, and technical problem solving. Websites, email, and instant messaging were the sources more frequently used and with higher satisfaction, than structured programs, social networks and multimedia content. Variability in the user profiles showed three clusters: Cluster 1 *Social network user* (*n* = 13), Cluster 2 *Diversified user* (*n* = 75) and Cluster 3 *Communicative instant user* (*n* = 60). Participants in Cluster 2 compared to those in the other clusters were the most proficient on their digital competences and acknowledge the positive impact of digital resources on their professional practice and the psychological and social wellbeing of families. This study points the need for improvement in professionals’ digital competences in some of the measured areas and the user profile of digital resources since both provide benefits on professional practice and family autonomy and wellbeing.

## 1 Introduction

Digital media is becoming a main part of all the facets in our lives from working to leisure or to communicate with each other’s. The recent COVID-19 pandemic has accelerated the trend toward the use of digital media and, at the same time, has evidenced the needs in terms of digital transformation, competences’ development and ethical challenges (Mishna et al., 2021; Pink et al., 2022; Fiorentino et al., 2023). At pace with these needs, the Digital Education Action Plan (2021–2027) aims to support the adaptation of the education and training systems of European Union member states to the digital age, based on standards of high-quality, inclusive and accessible digital education. The second priority set on this plan consists of enhancing digital competences in European countries, specifically in education and training areas.

Digital competences, according to the European Digital Competence Framework for Citizens (DigComp), are structured into five areas (Carretero et al., 2017): (1) information and data management, which involves skills related to organize, store and retrieve data, as well as to process them in a structured environment; (2) communication and collaboration, that deals with abilities to interact through a variety of digital technologies and to apply appropriate digital communication means to a given personal and societal context; (3) digital content creation, refers to create and edit digital content in different formats, besides to express oneself through different digital means; (4) safety measures, which is related to protective devices and digital content, to understand risks and threats in digital environments and to be responsible regard reliability and privacy; and (5) technical problem solving, referred to identify technical problems in the current digital tools and be able to solve them. For each area, users can show up from one to five proficiency levels: low, medium, intermediate, advanced, and specialized, considering the complexity of tasks, the autonomy in facing them and the demands involved.

The Child Protection and Family Support System is not oblivious to this spreading of information and communication technologies (ICTs). ICT tools are in increasingly used by practitioners for digital service provision, such as online assistance and therapy (individual, group and community dynamics), tailored advice through e-mail consultations, or carrying out the implementation and monitoring of online programs (Nieuwboer et al., 2013; Niela-Vilén et al., 2014; López-Peláez et al., 2017). The quick rise in the use of digital resources requires the developing of digital competences handled by professionals. However, the need for improvement in the use of digital resources coexist with the scant scientific evidence available on the current professional expertise in digital competences, the digital resources used by professionals and their impact on family autonomy and wellbeing. This is the topic of the present study applied to the field of child and family support delivered by social agencies.

The child and family support system has expanded since the Council of Europe’s Recommendation on family policies to support positive parenting (Council of Europe, 2006; Rodrigo, 2010). This initiative draws the attention to support the exercise of positive parenting in safeguarding the children’s rights, and the development of inclusive communities to make it possible. The child and family support system are usually settled on public social services and non-profit organizations or social agencies (NGOs). The latter occupy a relevant role in working with families since, in coordination with social services, they are usually in the frontline of the intervention with children, adolescents and families. Their work on family support is carried out by interdisciplinary teams, shaped by professionals such as social workers, psychologist, or social educators, among others. Traditionally, both social agencies’ and social services’ practitioners have been slow to adapt to technology (López-Peláez et al., 2017; Fiorentino et al., 2023). Despite this lag, the massive incursion of digital technology, especially from the health crisis of the last 3 years, have encouraged the digital transformation in social agencies and services. At the same time, Covid-19 pandemic has laid on the table the weaknesses of the family support system based on the professional use of digital resources for educational and intervention purposes.

Using ICTs in child and family support services imply advantages but also some risks or controversies (López-Peláez et al., 2017; Reamer, 2018; Suárez-Perdomo et al., 2018a; Mishna et al., 2021; Canário et al., 2022). On the one hand, it can serve as an incentive to encourage users to engage in the service. Besides, it allows gaining flexibility, accessibility and fluidity in the relationship between professionals and users. For example, technology has facilitated the reach of family or social education programs to remote areas. However, it carries out also some limitations such as the risk of dehumanize the relation with users (Reamer, 2018), the more difficulty to stablish a therapeutic alliance, or greater costs that may result in an increase of social exclusion for users who are not able to afford the devices or the access to online services (López-Peláez et al., 2017). Furthermore, the necessity to deal with ethical challenges and legal considerations in the use of ICTs is also a matter to consider. Digital ethics for therapists, as defined by the Zur Institute (2018), involves managing oneself ethically, professionally and in a clinically sound manner via online and in digitals mediums. Issues as ensure privacy and confidentiality, informed consent, protecting users’ data in computers, professional boundaries, managing records of online conversations or the responsibility to use online material from reputable sources should also be a matter of concern to social agencies’ practitioners (López-Peláez et al., 2017; Reamer, 2018; Mishna et al., 2021).

Despite of mastering digital competences is a requirement for these professionals in Europe, this subject has not been formally included in the training of social workers or others reference professionals (Taylor, 2017; Zhu and Andersen, 2022), although universities are increasingly investing in digital technologies that enable them to offer educational content in a more sustainable way and with a major impact on student well-being and assessment, this does not translate into digital skills training for their students (Panesi et al., 2020; Fülöp et al., 2022; Tripon et al., 2023). Baker and Hitchcock (2017) suggest that while social work students are using technology and social media in their everyday lives, they may not know how to use these tools appropriately in professional settings. In relation to ethical challenges, as Joiner (2019) has pointed, the lack of digital education and instruction focusing on the use of ICT in direct practice could result in future practitioners unable to address the ethical issues of the digital age. Therefore, an important component of ethical standards should be involved in training social services’ and social agencies’ practitioners about the use of technology in practice (Reamer, 2018).

Regarding the effect of ICTs on the users’ autonomy and wellbeing, following the model of parents’ well-being (Nelson et al., 2012) there are psychological mechanisms that mediate the relationship between parenthood and well-being, an adequate support system has been identified as one of the mechanisms related to higher family well-being. Nnabuko and Anderson (2018) found in their systematic review on the healthcare field that ICT interventions have a positive impact on the participants’ social network, especially when real-time interaction is also provided. The meta-analysis carried out by Nieuwboer et al. (2013) provides evidence for the effectiveness of the web-based interventions in supporting parents in their parenting role. Specifically, it is suggested that knowledge can be improved by self-guided web-based training programs, while positive changes in attitude and behavior may be obtained through internet interventions, intensively guided by therapists or coaches. The access to any kind of educational environment has not only an intrinsic value but also influences well-being indirectly by its impact in living conditions, healthier life-style, greater employability, education provides individuals with the skills necessary to integrate more fully into their societies (Sianesi and Van Reenen, 2003; Boarini and Strauss, 2010; Miyamoto and Chevalier, 2010; OECD, 2010, 2020). Hall and Bierman (2015) add that stronger effects in terms of both engaging parents and promoting positive outcomes for parents and children may emerge in blended intervention approaches that use technology along with synchronous communication support from professionals. In relation to the promotion of parents’ autonomy in the use of Internet, Suárez-Perdomo et al. (2018b) pointed that professionals have a responsibility in supporting parents to develop effectiveness abilities to navigate in Internet and finding reliable sources by themselves. Otherwise, the responsibility will lie exclusively on the parents who are not necessarily knowledgeable about the theories, contents, and practices they come across while browsing. Canário et al. (2022) also highlight that advice provided in online peer-led discussion groups that are not moderated by a skillful professional may be ineffective or even harmful. Despite this evidence, little is known about the practitioners’ digital competences in social agencies (NGOs), the variety of digital resources used by professionals and the impact on professional practice and family autonomy and wellbeing. To contribute to fill in this gap, this study aims to reach three objectives. First, to examine the level of professional expertise according with the five areas of digital competences normatively required by the European Digital Competence Framework for an effective, inclusive, and ethical use of digital resources in the field of child and family support. Second, to analyze the variety of user profiles given the broad range of digital resources available (e.g., websites, blogs, structured programs, instant messaging, videocalls, emails, multimedia contents, and social networks) measuring the frequency of use and usefulness. The diversity of use of digital resources creates different learning opportunities allowing professionals to build their own personal learning environment (PLE) defined as the “set of different resources that we used in our daily life to learn” (Attwell, 2007, p. 4). A person-centered approach (Bergman et al., 2003) was used to identify the interindividual variability in the digital user profiles. Third, to test the impact of level of competences and user profiles on four relevant outcomes: the actual improvements in the professional practice; family satisfaction with the services; child, and family wellbeing; and autonomy of the family in the exercise of the parenting role. Results will inform about the conditions in which professionals working in social agencies provide support to families through digital resources and their need for training to improve the quality assurance.

## 2 Method

### 2.1 Participants

Participants were 148 practitioners working in social agencies all over Spain. Most participants were females (78.4%) and had a mean of 36.8 years of age and a wide range of years of experience in child and family support with a mean of 11.2 years (see Table 1). The majority of them had a job as a frontline practitioner (56.6%) or coordinator (27.6%) and mainly work in Local and Regional NGOs. Professionals also work in bank (Caixa Proinfancia program) and professional foundations and cooperatives (28.1%).

**Table 1:** Participants description.

	*n*	Percentage	Mean	S.D.	Min. value	Max. value
Age			36.8	10.1	20	63
Years of experience			11.2	8.8	<1	36
Gender (female)	116	78.4				
Agency scope						
Local NGOs	67	45.9				
Regional NGOs	24	16.4				
National NGOs	10	6.8				
International NGOs	1	0.07				
Foundation/Cooperative	41	28.1				
Private company	3	2.1				
Job position						
Practitioner	82	56.6				
Coordinator	40	27.6				
Directive	23	15.9				

### 2.2 Survey content

According to the literature the survey was composed by four sections of questions: (a) demographic-professional data; (b) professionals’ digital competences in working with families; (c) use of digital resources and satisfaction; and (d) impact on professional and family variables. To facilitate the participants’ contextualization on the topic the order of the sections was: (a), (c), (b) and (d). A description of the content of each section follows.

(a) Demographic-professional data (five questions): age, gender, years of expertise in child and family support, type of agency (NGO, Foundation / Cooperative, and private company), job position (practitioner, coordinator, directive).
(b) Professionals’ digital competences in working with families (22 questions): based on the key five areas of the European Digital Competence Framework for Citizens (Carretero et al., 2017). The questionnaire is organized in five sections with good reliability in the current sample: (1) Information and data management involving navigation, search, data filtering and digital content, evaluation, storage and retrieval of information (five items, α = 0.861), (2) Communication and collaboration involving how to interact through technology, know how to share information and content at personal and citizen levels, as well as manage labels and identity (four items, *α* = 0.874), (3) Digital content creation involving the development of content and knowledge of copyright and licenses and integration re-elaboration of content (four items, *α* = 0.892), (4) Safety measures involving protection of devices, personal health and also safety for the environment, as well as the protection of personal data and privacy (six items, *α* = 0.894), and (5) Technical problem solving involving innovation and the creative use of digital technology, as well as the identification of gaps within digital skills (three items, *α* = 0.881). Answers were given on a five-point Likert scale ranging from Never to I can do it (1) and I can even help others (5).
(c) Use of digital resources (16 questions): measure frequency of use and perceived usefulness in websites, blogs, structured programs, instant messaging, videocalls, emails, multimedia contents (Youtube / Vimeo and Podcast) and social networks (TikTok, Instagram, Facebook, Twitter). The frequency of use was measured with a five-point Likert scale ranging from Never (1) to Everyday (1); and the perceived usefulness with a five-point Likert scale ranging from Non useful at all for my professional practice (1) to Absolutely useful for my professional practice (5).
(d) Impact of digital resources on professional and family variables: four aspects with one question each: (1) whether it is beneficial to improve my professional practice, (2) whether it is beneficial to improve family satisfaction with the service, (3) whether it is beneficial to promote psychological and social well-being of families, and (4) whether it is beneficial to promote the autonomy of families in the care and education of their children. Answers to each question were given on a five-point Likert scale of level of agreement ranging from Absolutely disagree (1) to Absolutely agree (1).

### 2.3 Survey administration and data collection

An advertising email on the project content, motivation for enrollment and future dissemination of results was sent to a list of local, regional, national, and international social agencies working in Spain. By using google forms the online survey was also sent and requested to be filled out in 2 months (from end of February to end of April 2023). A written informed consent form to participate and the use of the data anonymously for research, teaching and dissemination purposes was also included in the online survey (acceptance rate was higher than 80%). The data were exported to an excel file sheet automatically.

### 2.4 Statistical analyses

IBM SPSS Statistics Version 27.0 (Armonk, NY, IBM Corp) was used for the statistical analysis. For the first aim to examine the level of professional digital competence a descriptive analysis was used to identify mean scores and standard deviations. For the second aim to analyze the user profiles in a variety of digital resources, a descriptive analysis was performed showing the mean scores and standard deviations. Next, we analyzed the variability in the use of digital resources and their perceived usefulness using a two-step cluster analysis. First, a hierarchical analysis was performed to explore the initial setup and visual examination of the dendrogram, size of the clusters and theoretical interpretation. Second, an iterative non-hierarchical k-means cluster analysis with ANOVAs was performed to determine the significant variables that contribute to the solution. Third, univariate analysis of variance and chi-square test were performed to explore the differences between clusters, taking into account the Levene’s test results (equality of variance–*p* > 0.05), Scheffe post-hoc test was interpreted (Popa, 2010), and Cramer’s V was interpreted, since it is considered a robust test for strength of association within multiple group studies.

For the third aim to test the impact of level of competences and user profiles on four professional and family variables single-factor multivariate analysis of variance were performed, taking into account the Levene’s test results (equality of variance–p > 0.05), Scheffe post-hoc test was interpreted (Popa, 2010).

## 3 Results

### 3.1 Professionals’ expertise in digital competences

Our first aim was to explore the level of professional expertise in the five areas of digital competences, applying the equivalence of Likert scale scores to levels of competence: Low level (1 point); Medium level (2 points), Intermediate level (3 points), advanced level (4 points) and specialized level (5 points), according to the European Digital Competence Framework for Citizens (Carretero et al., 2017). Accordingly, Table 2 shows advanced levels in those competences related to Information and data management (e.g., organize, store, and retrieve data) and Communication and collaboration (e.g., to interact through a variety of digital technologies, to share digital content or to apply appropriate digital communication means to a given personal and societal context). Intermediate levels of competence were obtained in aspects that require more training and technological updating such as the Digital content creation and development, Safety measures such as protecting devices and secure management of personal data, and Technical problem solving such as creative use of digital technology.

**Table 2:** Level of digital competences in key five areas (1–5 scale).

Digital competences	M (SD)	Assigned level
Information and data management	4.09 (0.56)	Advanced
Communication and collaboration	4.13 (0.65)	Advanced
Digital content creation	3.62 (0.90)	Intermediate
Safety measures	3.65 (0.80)	Intermediate
Technical problem solving	3.64 (0.84)	Intermediate
Total mean of competences	3.82 (0.66)	Intermediate

### 3.2 Identifying user profiles of digital resources

The second aim was to explore the use of digital resources and the perceived usefulness of each of them for their work. Websites, email and instant messaging were the resources more frequently used by practitioners, whereas blogs and multimedia content were the less used, being structured family programs, social networks and videocalls monthly used (Table 3). Regarding perceived usefulness, emails and instant messaging are the tools perceived as the most useful, followed by structured family programs, videocalls and Websites. Instead, blogs, social networks and multimedia content were perceived as less useful.

**Table 3:** Use and usefulness of digital resources (1–5 scale).

Digital resource	Use *M* (*SD*)	Perceived usefulness *M* (*SD*)
Websites	4.51 (0.70)	4.29 (0.75)
Blogs	2.75 (1.07)	3.21 (1.12)
Structured programs	3.07 (0.81)	4.40 (0.75)
Instant messaging	4.80 (0.61)	4.63 (0.73)
Videocalls	3.22 (0.98)	4.31 (1.00)
Emails	4.83 (0.42)	4.78 (0.55)
Multimedia contents	3.00 (1.33)	3.42 (1.06)
Social networks	3.14 (1.50)	3.35 (1.14)

To identify individual differences in the user profiles of digital resources and the perceived usefulness of each of them for their work with families a hierarchical cluster analysis was performed showing three theoretically meaningful clusters in the use of digital resources and perceived usefulness. The hierarchical three-cluster solution was replicated using the iterative partitioning method, k-means, with squared Euclidean distance values between centers of clusters greater than 1 indicating a satisfactorily discriminating solution. The variables that contributed significantly to the clusters are presented in Table 4. Clusters are named according to the type of digital resources used: Cluster 1 *Social network user* (*n* = 13), Cluster 2 *Diversified user* (*n* = 75) and Cluster 3 Communicative instant user (*n* = 60). Post-hoc tests were conducted for significant differences among clusters.

**Table 4:** Cluster solution with variables and inter-cluster distance.

	C1. Social network user (*n* = 13)	C2. Diversified user (*n* = 75)	C3. Communicative instant user (*n* = 60)	*F* (2,145)	*Post-hoc test* Scheffe
Digital resources use					
Website	4.23	4.65	4.4	3.411*	1–2*
Blog	2.69	3.18	2.21	16.462***	2–3***
Structured programs	2.69	3.12	3.1	1.579	-
Instant messaging	3.84	4.85	4.95	23.150***	1–2*** 1–3***
Emails	4.15	4.89	4.91	24.957***	1–2*** 1–3***
Videocalls	2.38	3.3	3.3	5.436**	1–2*** 1–3***
Social networks	3.38	4.17	1.81	91.817***	1–2* 1–3*** 2–3***
Multimedia contents	3.46	3.82	1.88	69.621***	1–3*** 2–3***
Perceived usefulness					
Website	3.69	4.54	4.1	11.710***	1–2*** 2–3***
Blog	2.46	3.81	2.63	30.283***	1–2*** 2–3***
Structured programs	3.61	4.53	4.41	9.119***	1–2*** 1–3***
Instant messaging	3.23	4.76	4.78	39.086***	1–2*** 1–3***
Emails	3.76	4.89	4.86	35.056***	1–2*** 1–3***
Videocalls	2.53	4.58	4.35	32.804***	1–2*** 1–3***
Social networks	3.46	4.09	2.4	72.330***	1–2* 1–3*** 2–3***
Multimedia contents	3.53	4.05	2.61	51.452***	1–3*** 2–3***
Inter-cluster distance					
1	4.997	5.010	-		
2	3.331	-			
3	-				

*p ≤ 0.05; **p ≤ 0.01; ***p ≤ 0.001.

All clusters showed low levels of use of structured family programs, and they vary according to the use of other digital resources and the perceived usefulness of them. The Cluster 1 *Social network user* was represented by professionals with the highest rates in the use and perceived usefulness of social resources such as social networks and multimedia contents, and the relatively lowest levels of use and perceived usefulness of instant messaging, emails or videocalls. The Cluster 2 *Diversified user* was characterized by professionals with the highest rates in the use and perceived usefulness of all kind of digital resources, including static content, communicational and social resources. The cluster 3 Communicative instant user was characterized by professionals with high use and perceived usefulness of personal contact resources, such as instant messaging, emails and videocalls, and the lowest levels of use and perceived usefulness for their work of any other type of resources such as blogs, social networks, and multimedia contents.

### 3.3 Relationships between digital competences and digital user profile

Three of the five professional competences areas showed significant relationship with digital user profiles (Table 5). Those professionals with significantly higher levels of digital competences related to Information and data management, Safety measures and Technical problem solving skills are more likely to belong to the Cluster 2 Diversified user profile of digital resources. On the other hand, professionals who are less competent in the same areas were more likely to belong to the Cluster 1 Social network user profile and Cluster 3 Communicative instant user profile.

**Table 5:** Mean differences in the digital competences according to the digital user profiles.

Digital competences	C1. Social network user (*n* = 13)	C2. Diversified user (*n* = 75)	C3. Communicative instant user (*n* = 60)	*F* (2,145)	*Post-hoc* Scheffe
Information and data management	3.73	4.22	3.99	5.827**	1–2** 2–3*
Communication and collaboration	3.84	4.23	4.07	2.532	
Digital content creation	3.64	3.76	3.43	2.267	
Safety measures	3.28	3.79	3.54	3.246*	1–2*
Technical problem solving	3.41	3.8	3.49	2.947*	2–3*

*p ≤ 0.05; **p ≤ 0.01; ***p ≤ 0.001.

Professionals’ digital user profile is also related to professional characteristics. Those professionals belonging to Cluster 2 Diversified user profile of digital resources are significantly older (*M* = 38.56) and have more years of experience (*M* = 12.54), than those in Cluster 1 Social network user profile (*M* = 30.84; *M* = 6.46) (*F*(2,146) = 3.710; *p* ≤ 0.05; *η*^2^ = 0.05) (*F*(2,146) = 3.096; *p* ≤ 0.05; *η*^2^ = 0.04). In addition, professionals belonging to Cluster 1 Social network user profile were those who did not have any kind of specific training in child, adolescent and family care (*χ*2 (2, *N* = 148) = 9.828, *p* ≤ 0.01, Cramer’s *V* = 0.26).

### 3.4 Identifying impact of digital user profiles on professional, family wellbeing and autonomy dimensions

Professional digital user profiles are also related to relevant aspects in professional and family development. As is shown in Table 6, those professionals with a diversified use of digital resources profile are more likely to consider that the use of digital resources will improve their own professional practice and promote psychological and social well-being of families. On the other hand, the use of digital resources was not related to families’ satisfaction with the service or the promotion of their autonomy in the exercise of their parental role of childcare and education.

**Table 6:** Mean differences of the digital user profiles on professional and family outcomes.

	C1. Social network user (*n* = 13)	C2. Diversified user (*n* = 75)	C3. Communicative instant user (*n* = 60)	*F* (2,145)	*Post-hoc* Scheffe
Improve professional practice	4.07	4.50	4.23	3.834*	1–2* 2–3*
Improve family satisfaction with the service	3.53	4.06	3.93	2.426	-
Promote psychological and social well-being of families	3.15	3.88	3.50	5.569**	1–2** 2–3*
Promote autonomy of families in the parenting task	3.30	3.86	3.60	2.608	-

*p ≤ 0.05; **p ≤ 0.01; ***p ≤ 0.001.

## 4 Discussion

Regarding the first objective, results showed intermediate to advanced levels of professionals’ digital competences in the five areas. Results in Information and data management showed an advanced level of expertise, such as navigation, search, data filtering and digital content, evaluation, storage and retrieval of information, according to what is recommended by the European Digital Competence Framework. Responses also showed an advanced level with respect to the second area of Communication and collaboration that includes skills such as how to interact through technology, know how to share information and content at personal and citizen levels, as well as manage labels and identity, again according with the European Digital Competence Framework. Our results are in line with the finding that social services’ practitioners use digital skills for administration tasks and for communication in practice (Berzin et al., 2015). However, the above European framework also recommend a specialized skill level when it comes to the competence required to communicate with the citizens at large to disseminate results or to deliver prevention or promotion campaigns (Dolan et al., 2020). This is crucial in the current positive parenting policy framework with an emphasis in the development of inclusive communities to support families (Council of Europe, 2006; Rodrigo, 2010).

Concerning competences involving more technological aspects, such as digital content creation, safety measures and technical problem solving, our results showed that professionals have an intermediate level of competence, as it is recommended by the European framework. However, the European Digital Competence Framework also recommended that the integration and re-elaboration of digital content that are needed for designing and developing family intervention programs delivered by digital media should be at the advanced level. The design and delivery of family programs or family interventions also requires safety competences at advanced level, which ensure that interventions accomplish ethical standards regarding the protection of personal and users’ data and confidentiality (López-Peláez et al., 2017; Reamer, 2018; Mishna et al., 2021; Pascoe, 2022). Therefore, it seems that these more specific technical competences still need to be improved.

Concerning the use of digital resources, participants reported a frequently use of websites and emails for their professional practice. Besides, they consider these tools to be useful for their work. Emails are not only a tool for professionals’ communication, but also may be a way to complement in-person intervention through helping users to express themselves in-between sessions (Pascoe, 2022). Instant messaging-such as Telegram or Whatsapp-had a high level of use, though participants vary on their perceptions of usefulness. This variation may be related with ethical issues referred to setting boundaries between professional and personal live. Ubiquity of instant messaging makes it more difficult to separate both worlds, which may upset some professionals. In this sense, ethical standards should pay attention to why and how setting boundaries (Pascoe, 2022). In turn, online structured family programs show a lower level of use, despite the fact that practitioners consider these programs to be quite useful in the intervention with families. There has been a growth of digital family programs based on evidence, which have scientifically proved their effectiveness in family outcomes (Nieuwboer et al., 2013; Suárez-Perdomo et al., 2018a; Callejas et al., 2021). Despite this evidence, it seems that the use of these programs is not yet generalized in social agencies. Videocalls use seem that no longer have survived since the end of the COVID-19 pandemic. Its level of use is lower than other digital resources, though the perceived usefulness seems to remain higher. Dealing with online interventions carries out some challenges, such as creating a collaborative alliance with the user (Reamer, 2018). In light of these results, it seems that professionals still prefer in-person intervention whenever is possible. Finally, the use of more informal resources such as blogs, social networks or multimedia contents is less developed in social agencies. Yet, the supportive use of apps like podcast may improve treatment adherence and fidelity (Berzin et al., 2015), as well the use of blogs may support the coordination of professionals located in different regions (Sage, 2014).

The diversity of use of digital resources already mentioned, creates different learning opportunities allowing professionals to build their own personal learning environment (PLE; Attwell, 2007). This modality of e-learning has also emerged as an innovative approach for the promotion of parental capacities on child-rearing issues and for the provision of social support to parents (Ebata and Curtiss, 2017; Suárez-Perdomo et al., 2022). Using a person-centered approach (Bergman et al., 2003) three distinct user profiles were identified among professionals. The Cluster 1 *Social network user* (8.8%) involves professionals that frequently use social networks and multimedia contents in their practice, finding them useful. In comparison, they use instant messaging or videocalls less frequently than professionals in the other clusters. The Cluster 2 *Diversifier user* (50.7%) includes professionals who show the highest rates in the use and perceived usefulness of any kind of digital resources. These seem to be professionals who fancy digital media and consider they fit on their practice. The Cluster 3 *Communicative instant user* (40.5%) includes professionals who mainly use digital resources for communication purposes at person levels, including instant messaging, emails and videocalls. Interestingly, members of Cluster 2 with a richer personal learning environment perceived themselves as more skilful in digital competences as compared to the other two groups in three areas: information and data management, safety measures, and technical problem solving. In addition, it coincided with being older professionals with more years of experience in caring for children, adolescents and families. These professionals, from digital settings, are able to offer families not only external and innovative activities, but also involve them on regular basis in common, home-based and relatively accessible activities with family members, which are a great way to improve the well-being of parents and children (Zabriskie and McCormick, 2001; Fiese et al., 2002). On the contrary, the members of Cluster 1 with a more socialized learning environment are those younger and less years of experience, as well as, lower levels of specific training in child, adolescent and family care. These results accentuate the need for specific training, both in family care and in management, communication, safety and technical support of digital resources to be able to offer adequate support to families, mainly in professionals with little experience and training. The lack of digital education in the training of social services’ and social agencies’ practitioners, it is considered one of the most important challenges for optimal use of ICT in family support (Reamer, 2018; Joiner, 2019; Canário et al., 2022).

This study has also allowed to explore the impact of the digital expertise and user profiles on the professional and family outcomes. Participants in general consider that using digital resources had a positive impact on their professional practice and contribute to improve family satisfaction with the service, confirming their positive attitude toward ICT (Tóth and Jávor, 2022). However, the influence of digital resources on the promotion of psychological and social well-being of child and families as well as on the autonomy of families in the exercise of their parental role is less clear. It seems that professional awareness that developing ICT may also promote families’ well-being and autonomy is still a challenge that needs to be addressed (Berzin et al., 2015). Some progress in this direction is evident when comparing cluster results. Cluster 2 professionals characterized by a diversified use of digital resources compared to Cluster 1 and Cluster 3 professionals with a social and communicative use are more likely to consider that digital resources improve both their professional practice and the psychological and social well-being of families. Family support should be provided through appropriate family services, which should also enable the community to provide an appropriate social environment for families (Rodrigo et al., 2014), even digital environments, considering that well-being of families is clearly dependent on the well-being of the community at large (Daro and Dodge, 2009).

## 5 Limitations and recommendations

As for limitations, first we cannot claim that we have reached a representative sample of social agencies in Spain based on an online survey. However, the final profile of professionals who answered the survey on voluntary basis fits well with the workforce distribution of the social agencies operating in Spain. Secondly, due to the large number of questions in the survey we did not further explore the extent to which professionals support parents to navigate in Internet by themselves to foster their autonomy, which may be helpful to explore in future studies. Finally, the family outcomes could have been explored in more detail and using parents as informants.

This study presents evidence on the intermediate and advantage ability levels in digital competences reported by the professionals in the provision of support to child and families. The workforce distribution illustrates the existence of a rich network of social agencies close to local environments, which most benefits to families. We also provide evidence that encouraging the digital skill training can enlarge the opportunities to managing a broader set of digital resources, leading to a richer professional learning environment. Both, the training of skills and a broader user profile bring benefits on professional practice and family autonomy and wellbeing.

We propose the following recommendations based on our results. First, the need for improvement of professionals’ digital competences can be fulfilled through the investment of time and effort in the social agencies, along with the development of quality standards and protocols aimed at supporting the effective, inclusive, and ethical use of digital resources (Tóth and Jávor, 2022). There are three types of competences likely to raise their intermediate levels with formal training: (a) a specialized skill level for the competences required to communicate with the citizens at large to disseminate results or to deliver prevention or promotion campaigns; (b) an advanced skill level to integrate and re-elaborate digital content for developing and implementing online structured family intervention programs; and (c) an advanced skill level for safety concerns which ensure that interventions met ethical standards regarding the protection of personal data and confidentiality. Although new graduated or young professionals are expected to handle digital competences with more proficiency, this does not necessarily imply that they are ready to effectively implement digital tools into their professional practice (Berzin et al., 2015).

Second, regarding digital user profiles half of the participants had benefits from the use of a variety of digital resources since this profile was associated with a higher level of competences. That means that formal training should not be devoted to specializing professionals on the use of so call “best” digital resources. All of them may be necessary depending on the type of task to be performed in supporting child and families. Likewise, given the fast development in the ICT is better to train professionals to be updated with new tools that appeared and could be suitable for the work with families. Not only in times of crisis, but also as a quality mechanism to expand the number of resources and communicative actions to improve family care.

Third, the positive impact of digital resources on professional practice and family outcomes is yet to be fully discovered. Professionals should be aware of the benefits of using the digital resources bearing in mind their potential outcomes for themselves and for the work with families. Besides their benefits for the child and family wellbeing, it is important to consider the extent to which the use of digital resources may increase the family satisfaction with the support received and the autonomy of the family. On this latter regard, it is important to help families to find reliable sources by themselves for educational purposes to avoid the danger of being exposed to biased values and contents, poor e-learning environments and hidden commercial purposes.

Forth, support for the development of professional’s competence to use and create quality digital resources for the improvement of family support system should fall directly on policy makers in the areas of social welfare and education. It is necessary the development of social policies associated with the education and training of professionals in the social areas in digital competences, with the consequent investment in clear lines of funding in accordance with the European Digital Competence Framework for Citizens.

## Data availability statement

The raw data supporting the conclusions of this article will be made available by the authors, without undue reservation.

## Ethics statement

The studies involving humans were approved by the Ethics Committee of the University of La Laguna, Spain (CEIBA2021-3114) The studies were conducted in accordance with the local legislation and institutional requirements. In accordance with the Declaration of Helsinki, written informed consent was obtained from the participants.

## Author contributions

SB: Data curation, Formal analysis, Methodology, Project administration, Writing – original draft, Writing – review & editing, Supervision, Validation, Visualization. GR: Data curation, Methodology, Supervision, Validation, Visualization, Writing – original draft, Writing – review & editing. MÁ: Data curation, Methodology, Validation, Visualization, Writing – review & editing. NG-R: Data curation, Validation, Visualization, Writing – review & editing. MR: Conceptualization, Investigation, Methodology, Project administration, Supervision, Validation, Visualization, Writing – original draft, Writing – review & editing. SP: Data curation, Validation, Visualization, Writing – review & editing. JM: Validation, Visualization, Writing – review & editing.
